# Learning to classify species with barcodes

**DOI:** 10.1186/1471-2105-10-S14-S7

**Published:** 2009-11-10

**Authors:** Paola Bertolazzi, Giovanni Felici, Emanuel Weitschek

**Affiliations:** 1 Istituto di Analisi dei Sistemi e Informatica "Antonio Ruberti", Consiglio Nazionale delle Ricerche, Viale Manzoni 30, 00185, Rome, Italy

## Abstract

**Background:**

According to many field experts, specimens classification based on morphological keys needs to be supported with automated techniques based on the analysis of DNA fragments. The most successful results in this area are those obtained from a particular fragment of mitochondrial DNA, the gene cytochrome c oxidase I (COI) (the "barcode"). Since 2004 the Consortium for the Barcode of Life (CBOL) promotes the collection of barcode specimens and the development of methods to analyze the barcode for several tasks, among which the identification of rules to correctly classify an individual into its species by reading its barcode.

**Results:**

We adopt a Logic Mining method based on two optimization models and present the results obtained on two datasets where a number of COI fragments are used to describe the individuals that belong to different species. The method proposed exhibits high correct recognition rates on a training-testing split of the available data using a small proportion of the information available (e.g., correct recognition approx. 97% when only 20 sites of the 648 available are used). The method is able to provide compact formulas on the values (A, C, G, T) at the selected sites that synthesize the characteristic of each species, a relevant information for taxonomists.

**Conclusion:**

We have presented a Logic Mining technique designed to analyze barcode data and to provide detailed output of interest to the taxonomists and the barcode community represented in the CBOL Consortium. The method has proven to be effective, efficient and precise.

## Background

In this paper we consider an automatic data analysis method to perform the classification of specimens through the analysis of a small portion of the genetic information extracted from specimens. The method proposed in this paper does not address the counterpart of this problem, i.e. the problem of clustering a collection of DNA sequences into groups which could potentially correspond to biological species; such approach has been widely adopted in many related papers discussed below and is frequently based on the search of tree-like structures that would be able to convey also the taxonomic relations among the examined species. In [1] a wide discussion of the two problems and the benefits and drawbacks in using DNA sequences is addressed.

Specimens classification methods based on a small DNA subsequence are first proposed for least morphologically distinguished species like archaea, bacteria, protists and viruses [2-4] and then extended to higher organisms [5,6].

In his first paper on this topic [7] Hebert uses *DNA Barcoding*, a technique based on a short DNA sequence from a small portion of the mitochondrial DNA (mt-DNA), the gene cytochrome c oxidase I (COI), to be used as a taxon "barcode", that differs by several percent, even in closely related species, and collects enough information to identify the species of an individual. This molecule, previously identified by [8] as a good target for analysis, is easy to isolate and analyze and it has been shown [9] that it resumes many properties of the entire mt-DNA sequence. Since 2003 COI has been used by Hebert to study fishes, birds, and other species [10,11]; one of the most significant results concerns the identification of cryptic species among insect parasitoids [12]. For sake of completeness we remind that another mt-DNA subsequence (gene), Cytochrome b, was proposed as a common species-level marker, while COI is specific for animal species [13].

On the basis of these results the Consortium of Barcode of Life (CBOL) was established in 2004. CBOL is an international initiative devoted to developing DNA Barcoding as a global standard for the identification of biological species, and has identified data analysis issue as one of the central objectives of the initiative. In particular the Consortium proposed several key problems:

1. Optimize sample sizes and geographic sampling schemes, as barcodes are not easy to measure, and large samples are very expensive;

2. Consider various statistical techniques for assigning unidentified specimens to known species, and for discovering new species;

3. Stating similarity among species using character-based barcodes and identify what are the character based patterns of nucleotide variation within the sequenced region;

4. Identify small portion of the barcode that are relevant for species classification, as sequencing long molecules is expensive (shrinking the barcode).

In this paper we deal with the last two items. We propose a method that, given a sample, finds a small relevant portion of the COI sequence that allows to distinguish among the species that are present in the sample, and we provide a character based pattern for each species (i.e. a *logic formula*) that allows to precisely classify all the individuals of the sample and individual whose species is unknown (partially addressing also the second item of the list). The method, already described and applied in some other variants in previous work [14-17], is new for this problem and does not provide explicitely a taxonomic structure of the analyzed species.

The topic of shrinking the barcode is studied in [18], where the authors determine how much sequence information is required for identification and calculate the probability of having species-specific barcode for varied size fragments. They show that sequences of among 100 and 250 sites are most of the time sufficient. In [19] it is shown that while long sequences are needed to obtain correct phylogenetic trees and to identify new species, smaller sequences are sufficient to classify specimens.

Most of the known methods for barcode analysis are either based on the concept of *distance *between M-OTUs (*Molecular Operational Taxonomic Units*), or *character based*. An M-OTU is a terminal node (an organism) in coalescent trees obtained by sequencing an informative sequence of DNA. Among the distance based methods we recall that one where M-OTUs are analyzed by first creating M-OTU profiles (i.e. identifying those sites where two unrelated individuals are unlikely to have the same alleles) and then using the Neighbor Joining (*NJ*) method [20] to obtain a phylogenetic tree (the NJ tree), so that each species is identified as represented by a distinct, non overlapping cluster of sequences in this tree. The principle of the NJ tree is to find pairs of M-OTUs that minimize the total branch length at each stage of clustering of M-OTUs starting with a star-like tree. A model-based, decision-theoretic framework based on the coalescent theory, where both distance and the posterior probability of a group are utilized is presented in [21]. Finally, in [22] simulations to test the performance of different methods based on sequence comparison (BLAST and Genetic distance) are described.

Among character-based methods, we recall here the method due to Kuksa and Pavlovic [23] and the one proposed by Sarkar et al. [24,25]. In [23] string kernel methods for sequence analysis are applied to the problem of species-level identification based on short DNA barcodes. This method does not require DNA sequences to be aligned; sorting-based algorithms for exact string *k*-mer kernels and a divide-and-conquer technique for kernels with mismatches are proposed. Similarity kernel allows to build accurate predictors and to cluster unknown sequences. The Characteristic Attribute Organization System (CAOS), proposed in [24,25], is a method for discovering conserved character states from cladograms (i.e., trees) or groups of categorical information. CAOS identifies character states at each node in a phylogenetic tree, in a similar way that attribute tests are identifies in decision tree algorithms. The method first identifies diagnostic DNA sequence changes in a data set, and then establishes those as rules for the second function of the program that reads DNA sequences from query specimens and assigns them to their species. Other character based methods use neural network [26] or Bayesian statistics [27].

The method proposed in this paper is a character based method and is comprised of two steps. The first step is feature selection, where the problem of selecting a small number of relevant features is formulated as an integer programming problem (a similar approach for feature selection has been also adopted in [17]). The second step is the identification of the logic formulas that separate each class from all the others. Such task is accomplished using the *Lsquare *system for logic mining, originally described in [14].

The main benefit of this method with respect to other more standard data mining approaches is its capability to provide compact classification rules that possess semantic information, since they identify, for each species, the sites of the molecule, the alleles that are characteristic of that species, and the propositional logic formulas that link them.

The paper is organized as follows: after a brief introduction to the notation and definitions used in the paper, in section "Shrinking the barcode", we describe the features selection model adopted, while in section "The extraction of separating logic formulas" we provide the reader with a synthetic description of the logic mining method *Lsquare *(further details are found in the related literature). Then, in section "Results and discussion" the data set used and the results of the experiments are described. Final remarks are the topic of section "Conclusions".

## Methods

We introduce the terminology adopted in the paper. We assume that each individual (or specimen) is described by its barcode, that in turn is composed of a fixed number of *m *sites (approx. 650, in the case of COI). Each individual belongs to one and only one species, or *class*. The data set is composed of *n *individuals, belonging to two or more classes; we refer to the sites of the barcode also as *features*, when we are in a mathematical setting. The *i *- *th *individual of the data set is represented by the vector *f*_*i *_= (*f*_*i*1_, *f*_*i*2_,..., *f*_*im*_), where *f*_*ij *_∈ {*A*, *C*, *G*, *T*}; the data matrix is represent by the sequence of vectors *f*_1_, *f*_2_,..., *f*_*n*_. Given this matrix representation of the data set, when appropriate the individuals may also be referred to as *rows*, while the features as *columns*. The classification method adopted is basically a two-class separation method, in the sense that it identifies the logic formulas that separate the individuals of one class in the data set from the remaining individuals of the data set (such individuals may belong to one or more classes). When needed, we refer to the two classes to be distinguished as *class A *and *class B*.

### Shrinking the barcode

The identification of a subset of relevant features among a large set is a typical problem in Data Analysis and Data Mining, often referred to as *feature selection*. Among the different approaches, the idea of formulating the feature selection problem as a mathematical optimization problem where the number of selected features is to be minimized under some constraints has received some attention in the literature, and proven to be effective in many situations. In [28] the authors adopt such an approach for the selection of TAG SNPs; the mathematical model adopted turns out to be a linear problem with binary variables whose structure is well known in the combinatorial optimization literature as the *set covering problem*. Several similar models where also treated in [29], where large set covering models where proposed (a.k.a. the *test cover *problems). The main drawback of this approach, and of the many variants that have been then proposed, lays in the fact that it uses one constraint of the integer programming problem for each pair of individuals of the data set that belong to different classes. Such fact implies a rapid growth of the dimension of the problem, and thus its intractability for large sizes, that then requires the use of non optimal solution algorithms.

In this paper we adopt an alternative approach, characterized by:

a. the use of a different method to construct the constraints of the optimization problem, that results in a problem size that grows only linearly with the size of the data;

b. the use of a different objective function, that maximizes a lower bound on the discriminating power of the solution over all the individuals of the training data.

Such formulation can be solved in reasonable time with exact optimization algorithms, but very good quality solution can be found also with fast heuristics developed ad hoc; this is the case of the GRASP method used in this paper and described in [17].

Such approach is based on a very simple idea.

For the time being, we assume the individuals to belong to only two classes, class A and class B. Given a feature *f*_*j*_, we define *P*_*A*_(*j*, *k*) and *P*_*B*_(*j*, *k*) be the proportion of individuals where feature *f*_*j *_has value *k *(for *k *∈ (*A*, *C*, *G*, *T*)) in sets *A *and *B*, respectively. If *P*_*A*_(*j*, *k*) *> P*_*B*_(*j*, *k*) (resp. *P*_*B*_(*j*, *k*) *> P*_*A*_(*j*, *k*)), then the presence of *f*_*j *_with value *k *is likely to characterize individuals that belong to class *A *(resp. *B*). To better qualify the strict inequality between *P*_*B*_(*j*, *k*) and *P*_*A*_(*j*, *k*) we introduce an additional parameter *λ *> 1, and then define, for each feature *j *and for each individual *i *in class *A *the vector *d*_*ij *_as follows.



While, for individuals *i *in class B, the value of *d*_*ij *_will be:



In the practical application the parameter *λ *directly influences the density of the matrix composed of *d*_*ij *_and can be adjusted to obtain a reasonable value for the density itself (say 20%).

According to this definition, we intuitively assume that the number of ones in vector *d*_.*j *_is positively correlated with the capability of feature *f*_*j *_to discriminate between classes A and B. We would then like to select a subset of the features that exhibits, as a set, a good discriminating power for all the individuals considered, so that we may use more features combined together to build rules that perform a complete separation between *A *and *B*.

The purpose of the feature selection model is then to select a given and small number of features that, collectively, guarantee a good discriminating power for all the individuals of the data sets. This can be formally stated asking to select a given number of features (say, *β*) that maximize the minimum of the discriminating power over all the individuals.

We define the binary decision variable *x*_*j *_= {0, 1} with the interpretation that *x*_*j *_= 1 (resp. *x*_*j *_= 0) means that feature *j *is selected, (resp., is not selected). The binary integer optimization problem can then be defined as follows:

(1)

We bring to the attention of the reader the fact that *β *is a parameter of the problem, and not a variable. The optimal solution of the above problem would then select the *β *features that guarantee the largest discriminating power over all the individuals in the data. Despite the problem has been described with straight-forward arguments, it can easily be shown that its optimal solution amounts to identify the feature set of a given size that maximize the additive *class entropy *of its individuals. Besides, the number of variables of the problem is given by the number of features (*m*), and the number of rows by the number of individuals (*n*), keeping the size of the problem in a linear relation with the size of the data. The problem is anyway difficult to solve, and for large sizes approximate solution methods may be needed if one is not to resort to heavy and often expensive commercial solvers for integer programming. The use of an efficient heuristic method allows the current implementation of the methods described in section "The BLOG software system" to be freely distributed for the purposes of the CBOL Consortium.

Once an optimal set of *β *features is selected, these are used by the logic mining tool *Lsquare *to extract the separating formulas, as described in the next section.

### The extraction of separating logic formulas

*Lsquare *is a learning method that operates on data represented by logic variables and produces rules in propositional logic that classify the individuals in one of two classes. The appropriateness of *Lsquare *for specimens classification is motivated by the fact that it uses a logic representation of the description variables, that are to all extents logic variables, and of the classification rules, that are logic formulas in Disjunctive Normal Form (DNF). Such property enables to analyze the classification results also from the semantic point of view, as the classification rules determined by the method express combination of the features that can be appreciated by domain experts and may bring to light useful knowledge in an easily understandable format.

The classification rules are determined using a particular problem formulation that amounts to be a well know and hard combinatorial optimization problem, the *minimum cost satisfiability problem*, or MINSAT, that is solved using a solver based on decomposition and learning techniques [30]. The DNF formulas identified have the property of being created by conjunctive clauses that are searched for starting from those that cover the largest portions of the training set. Therefore, they usually are formed by few clauses with large coverage (the interpretation of the trends present in the data) and several clauses with smaller coverage (the interpretation of the outliers in the training set).

From the practical standpoint, the problem of finding a separating DNF for *A *and *B *is solved sequentially, identifying at each iteration a conjunctive clause that holds *True *for the largest non-separated subset of *A *and *False *for all *B*. Termination of the process is guaranteed by some property of the method (see [14]). Each iteration is in turn formulated as a logic optimization problem, that we briefly describe here. Basic notions of propositional logic are given for granted and can be found in [14]; below we summarize the two main steps of the method.

First, we expand the selected features into 4 different logic variables, each one associated with the presence or absence of one the 4 nucleotides in the given position. For example, *v*_*jA *_with value *True *indicates that in position *j *is present nucleotide *A*, and *False *otherwise. For simplicity, assume that all these logic variables are sequentially indexed from 1 to *M*, and referred to as *v*_*j*_. Thus, *v*_*j *_= *True *for individual *i *means that, for that individual, a certain position exhibits a certain nucleotide.

Second, we formulate a MINSAT problem whose solution identifies one of the CNF clauses that will form the final DNF formula. To do this, we introduce two additional types of logic variables:

• *p*_*j *_and *q*_*j*_, linked with the *v*_*j*_s as follows: *v*_*j *_is chosen in the clause with value *True *if *p*_*j *_= *True *and *q*_*j *_= *False*; *v*_*j *_is chosen in the clause with value *False *if *p*_*j *_= *False *and *q*_*i *_= *True*, and *v*_*j *_is not chosen in the clause if *p*_*j *_= *q*_*j *_= *False*;

• *e*_*i*_, associated with each individual *i *of class *A*, that are forced by the constraints to assume value *False *if the clause identified by the solution holds *True *for *i*, and *False *otherwise.

Also, define as  the set of indices of the features that appear in individual *i *of class *A *with value *True*; and, symmetrically define as  the set of indices of the features that appear in individual *i *of class *A *with value *False*. Analogously define  and .

Consider now the following MINSAT problem, whose solution is determined by an assignment of the logic variables *p*_*j *_and *q*_*j *_and *e*_*i *_such that all the logic constraints are satisfied and the sum of costs of the variables that hold *True *is minimized:

(2)

It can be verified that the solution of problem (2) identifies a CNF clause on the *v*_*j *_and that the set *A' *= {*i *∈ *A*|*e*_*i *_= *False*} is the largest portion of *A *that can be separated from *B *by a simple CNF clause. In fact, we have that: *v*_*j *_belongs to the clause with value *True *if *p*_*j*_= *True *and *q*_*j *_= *False*; *v*_*j *_belongs to the clause with value *False *if *p*_*j *_= *False *and *q*_*j *_= *True*; and, finally, that *v*_*j *_is not present in the clause if *p*_*j *_= *q*_*j *_= *False*. Using this information, we can formulate a second MINSAT problem where we select, amongst all separating clause that separate *A' *from *B*, the one that uses the least number of literals (e.g., the most compact clause):

(3)

A more detailed description of the system and of its other components can be found in the related papers [14-16].

### The BLOG software system

The above mentioned methods have been integrated in a data mining software designed specifically for barcode analysis applications, the BLOG system (Barcoding with LOGic formulas).

The aim of the system is to identify logic rules that are able to recognize the species (in the following also referred to as *class*) of a specimen by analyzing its barcode sequence. A consistent effort in developing BLOG has been devoted to the import of files from different data format, to the control of the analysis flow, and to the production of output that are in line with the typical styles and interests of the scientific communities that use barcode or, more in general, DNA and RNA data, to extract classification rules from training data and to assess their quality.

The standard input of the program is a FASTA format file of barcode sequences containing the training and the testing set. The FASTA format is an internationally agreed upon format for nucleotide or peptide sequences. It is converted into an internal format named DMB that provides all the information needed for the analysis in an efficient format specifically designed for the adopted algorithms. In DMB, the data set is represented by a set of files each containing a specific portion of the complete information; each file is preprocessed, and a coherency and integrity control step on the data is performed before the real analysis starts. The main files that compose the DMB format are four, and contain respectively the data matrix (file *.matrix*), the class label and textual description for each specimen (file *.classes*), the identification number and the textual label for each column or locus (file *.collabels*) and, finally, the code and the description of each specimen (file *.rowlabels*).

The main outputs of BLOG are:

• The predicted class for each specimen with attached a measure of the confidence associated with such prediction;

• For each class in the training set, the logic formulas that have been extracted from data and are used to predict the class of the specimens; each formula is completed with a measure of its accuracy;

• The usual classification statistics (confusion matrix, average and variances of error rates obtained with different sampling strategies).

Additional output used to interpret the results is also provided:

• The name of each specimen in the test set followed by a list of the similar specimens in the training set and the accuracy measure of the classification of the test specimen (file *.bdp1*). For example, the following line:

EF539303; DQ108243, DQ108244, DQ108245, DQ108246, DQ108251, EF539302, DQ108252; 100%.

declares that the specimen with code EF539303 in the test set is recognized to belong to the same class as the specimens DQ108243, DQ108244, DQ108245, DQ108246, DQ108251, EF539302, and DQ108252; besides, the logic formulas that assert such similarities are able to classify all the test individuals of this class without error (with precision 100.0%).

• The logic formulas associated with each class; a sample of such output is given in Table 1 below, where the formulas associated with 4 species are described. The formulas are formed by a limited number of statements that identify the value (A ,C, G, or T) that the given position of the barcode needs to have to satisfy the formula; the following *Coverage *indicates the number of specimens recognized by that formula.

**Table 1:** Logic Models Extracted from Data

**SPECIES**	**CLAUSE(S)**	**COVERAGE**
Lonchofilla thomasi	v83 = t	19
Molossus molossus	v344 = t	11
Rhinofilla pumilio	v64 = t	20
Sturnira tildae	v616 = g	8

The BLOG system has been developed using a *Pipes and Filters *architecture [31], which provides a structure for computing streams of data. Each computational analysis or data manipulation is done by a separate module, or *filter*. The new data is transferred through pipes between the connected modules. The basic computational modules of the system have been written in ANSI C, while a high level web interface can be used to design and interact with the analysis stream. It runs both under Windows and Linux environments.

**Figure 1:**
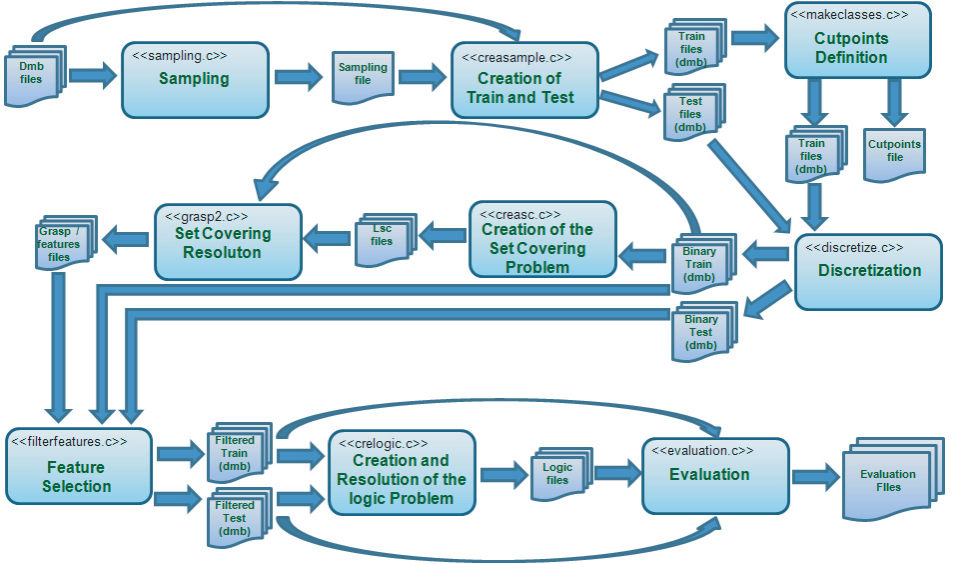
**BLOG flow chart**. The flow chart of the BLOG software system.

The flow diagram of BLOG in Figure 1 shows a schematic view of the architecture by representing the system flows and the fundamental modules.

We provide below a description of the BLOG modules:

• The *Sampling *module divides the original data files into two or more random samples, according to the sampling strategy that is requested by the user. The system implements standard Percentage Split and Cross-Validation; the sampling is designed to take into account, if required, the class distribution, and to perform a required number of repetitions of the sampling step. The created samples are stored in an incidence matrix with the individuals on the rows and the samples on the columns, that is then passed to the next module.

• The *Cresample *module starts from the sampling matrix and creates the required samples in an expanded DMB format to be used by the following modules; when more samples are to be analyzed, this module is called within a loop.

• *Makeclasses *is then used to identify the different values that each column may assume. This step is of particular importance when the data is in numerical form, and thus it is required to properly define, for each variable, two or more intervals by means of which a numerical variable can be considered a nominal, or logic, one. For the barcode application the identification of the value is straight-forward, as they will correspond to the 4 allelic values that can appear on the sites of the barcode, i.e., A, C, G, and T. Nevertheless, this module allows for the introduction and the proper processing of missing values, when present.

• The *Discretize *module is in charge of creating a new data matrix where each original value is mapped into a numerical code representing the corresponding interval, or value, that appears in the original matrix.

• The *Creasc *module takes as input the training file in DMB format and creates the optimization problem described in section "Shrinking the barcode".

• The *Grasp *module then executes the Grasp solution algorithm (see for reference [17]) to solve, for a given value of *β*, the optimization problem created by the *Creasc *module. The solution of the problem consists in a list of the *β *features selected.

• The *Feature Selection *then projects the samples from their original space into the new, smaller space, defined by the selected features. Such projection affects in the same way the training and the testing samples.

• The *Crealogic *module creates the MINSAT problem described in section "The extraction of separating logic formulas" in the specific input format required by the logic solver *Lsquare*, that is then called for each different species to create the classification logic formulas.

• The final module, called *Evaluation*, integrates all the results that have been obtained, in each experiment, by different loops over the classes and over the repetition of the experiments, and creates the output files for the final interpretation of the results.

A standard application of BLOG is structured in the following way. For each species, or class, *s*, a 2-class classification problem is defined, where class *A *contains the individuals of species *s*, and class *B *the individuals of the other species. The training data is used to formulate the feature selection problem described in section "Shrinking the barcode", and to identify the optimal set of features for different values of the parameter *β*. The *Lsquare *system is used to identify logic formulas based on the selected features, to separate the individuals in class *A *from those in class *B*. The formula for species *s *is saved, and the above is iterated for all the species. At the end of this process, we have the logic formulas - one for each class - and apply these formulas to the individuals of the training and testing splits. If an individual is recognized as positive by the formula of species *s*, we declare its predicted class to be *s *and then verify if such prediction is correct. When an individual is recognized as positive by more than one formula (or by none of them) we register such an event as a recognition error.

## Results and discussion

The software has been tested on two data sets provided by the Consortium for the Barcode of Life and one obtained directly from the GenBank Nucleotide Database.

The first data set was provided by the Consortium for the Barcode of Life in the 2006 Conference; it is composed of 1700 barcode sequences coming from individuals belonging to 150 different species. Each fragment contains between 648 and 690 sites (or nucleotides). Each sequence is extended to 690 sites for reaching the same length; initial and final gaps, if present, are handled by our algorithm (the special character 0 is used). This is a selection of barcode datasets extracted from BOLD (Barcode Of Life Database), which is based on real barcodes. The species are intentionally hidden by the Consortium and are only denoted by an incremental number. The choice of this data set is motivated by the following arguments:

• The species are not equidistributed: every species is represented, on the average, by 10 specimens, but there are some classes with limited available specimens, that make the problem harder (small training subsets);

• The variety of data from many species makes it hard to identify the specimen class.

The experiments have been conducted according to the previous described scheme (2-class classification problem):

• The data is split into training and testing data, adopting a proportion of 80% or 85% or 90% as training (the remaining being used for testing);

• For each species *s*:

- a 2-class classification problem is defined, where class *A *contains the individuals of species *s*, and class *B *the individuals of the other 149 classes.

- the training data is used to formulate the feature selection problem described in section "Shrinking the barcode", and to identify the optimal set of features for different values of the parameter *β *(10, 15, and 20).

- The *Lsquare *system is used to identify logic formulas based on the selected features, to separate the individuals in class *A *from those in class *B*.

At the end of this process, we have 150 logic formulas - one for each species - and apply these formulas seperately to the individuals of the training and of the testing splits.

The scheme is then repeated for different random splits of the data in training and testing.

The results are summarized in Table 2, that reports a row for each of the experiments that have been conducted. The values of *β *(10, 15, or 20) and the corresponding value of *α *obtained from the optimal solution of the feature selection problem are listed in columns 1 and 2; column 3 contains the percentage of data used for testing (10% or 15% or 20%). In the last 2 columns are reported the percentage of error obtained on the training and on the testing data, respectively.

**Table 2:** Optimal values and Error Rates (first data set)

			**Error Rates**	
***β***	***α***	**test%**	**training**	**testing**
10	4	10	8.02%	17.00%
10	4	10	10.14%	20.00%
10	4	20	11.90%	21.52%
10	4	20	13.50%	21.19%

average			10.89%	19.93%

15	6	10	0.87%	10.00%
15	6	10	1.93%	12.50%
15	6	20	1.50%	10.93%
15	6	20	2.04%	12.25%

average			1.58%	11.42%

20	8	15	0.20%	8.94%
20	8	15	0.61%	7.72%

average			0.40%	8.33%

The overall error rate decreases, as is to be expected, when a larger training set is used, due to the fact that the information used to extract the formulas is larger and the formulas are therefore more accurate. In the same way, we note that experiments with fewer features (where *β *= 10) are less precise than those with more features; to any extent, for the largest values of *β *used (20), the error rates are very small also when the testing data used is larger (20%). This means that the system is able to extract good formulas using only 20 of the 648 sites that are present in the barcode. Moreover, when comparing the error rates obtained on the training set with those obtained on testing we note a very little decay in the performances, thus highlighting the good generalization capabilities of the formulas and the important role of the barcode data for species discrimination.

It is of interest to check the frequency by which the different features (barcode sites) appear in all the formulas that have been identified for the different random splits. We identify a group of sites that appear with particularly high frequency (i.e., are present in many of the formulas obtained by the method) that are likely to be those whose combination best expresses the difference among the 150 species considered: such sites are in position 100, 106, 470, 469, 544, 637, 331 of the barcode.

The logic formulas are indeed very compact, and very few of them are composed of more than one CNF clause; such clauses are composed of few (usually 3, but never more that 5) literals (i.e., combination of a feature and its value). In Table 3 we report as an example a list of the separating formulas for the first 5 species of the 150 available for one of the experiments with the corresponding correct coverage (cc) and wrong coverage (wc):

**Table 3:** Logic Formulas for Species 1 to 5 (first data set)

**SPECIES**	**CC**	**WC**	**CLAUSE(S)**
A1	1.00	0.00	(v100 = c) and (v346 = a) and (v499 = t) and (v502 = a)
A2	0.77	0.00	(v82 = t) and (v238 = t) and (v502 = c)
A3	1.00	0.00	(v58 = a) and not(v100 = c) and not(v106 = a)
A4	1.00	0.00	(v106 = t) and (v139 = g)
A5	1.00	0.00	not(v106 = g) and not(v295 = a) and not(v295 = g)

The example of the interpretation of the formulas for a subset of the analyzed classes is given in Table 3:

• First line of Table 3: *if nucleotide in position 100 of the barcode has value C, nucleotide in position 346 has value A, nucleotide in position 499 has value T and nucleotide in position 502 has value A then the species is 1 with correct coverage of 100% and wrong coverage of 0%*.

• Second line of Table 3: *if nucleotide in position 82 of the barcode has value T, nucleotide in position 238 has value T and nucleotide in position 502 has value C, then the species is 2 with correct coverage of 77% and wrong coverage of 0%*.

The second data set was provided by the Consortium for the Barcode of Life and is composed of 826 barcode sequences coming from specimens belonging to 82 different species. The barcode sequences are mainly taken from the Kingdom Animalia, the Phylum Chordata, the class Mammalia, the Infraclass Eutheria, the

Superorder Laurasiatheria and the Order Chiroptera, or commonly know as bats. Also in this case, each sequence is extended to 660 sites (or nucleotides) with special handling for gaps, if necessary. The sequences are taken from BOLD (Barcode Of Life Database) and differ in variety of aspects, length and composition. For this data set the class distribution is more fair, with 10 specimens for each, on the average. We have chosen this data set to have a comparison between a fair distributed and a more spread and less populated set.

The experiments have been conducted according to the previously described scheme.

As shown in Table 4 the results for a less populated set with fewer species have an increased recognition rate. When focusing on a given experiment, interesting considerations may be drawn on the results by a more detailed analysis. For example, considering the correct recognition rates on test individuals for the different classes, we note that, out of the 82 species only 3 classes do not have a perfect classification rate; namely, *Noctilio_albiventris_PS1*, *Phyllostomus_discolor*, and *Eumops_hansae*. These 3 classes are quite under-represented in the training data (with 3, 3, and 6 individuals respectively) and this may easily be a reason for the poor performances obtained; moreover, we also note that for the first 2 species no individual in the test data is recognized correctly, and only 50% in the third. Such type of information may easily indicate a structural similarity of these 3 species with other species that are present and more richly represented in the training data. An example of the logic rules associated with a subset of the species is reported in Table 5.

**Table 4:** Optimal values and Error Rates (second data set)

			**Error Rates**	
***β***	***α***	**test%**	**training**	**testing**
10	8	10	15.47%	15.06%
10	8	10	16.48%	15.90%
10	7	20	19.97%	21.03%
10	7	20	21.39%	22.56%

average			18.32%	18.64%

15	11	10	5.52%	6.67%
15	11	10	6.37%	8.33%
15	11	20	7.40%	10.42%
15	11	20	10.88%	10.42%

average			7.54%	8.96%

20	14	10	0%	1.38%
20	14	10	2.22%	3.08%
20	15	20	1.97%	5.50%
20	15	20	1.58%	5.13%

average			1.44%	3.77%

**Table 5:** Logic Formulas for Species 1 to 5 (second data set)

**SPECIES**	**CC**	**WC**	**CLAUSE(S)**
Ametrida centurio	1.00	0.00	(v182 = g) and (v290 = g)
Anoura caudifer	1.00	0.00	(v83 = c) and (v416 = a) and (v470 = c)
Anoura geoffroyi	1.00	0.00	(v290 = g) and (v416 = a) and (v470 = t)
Anoura latidens	1.00	0.00	(v266 = t) and (v377 = c) and (v416 = a)
Artibeus amplus	1.00	0.00	(v140 = t) and (v473 = t) and (v512 = c) and (v602 = c)

The third data set was obtained from GenBank Nucleotide Database and is composed of 626 recent barcode sequences coming from specimens belonging to 82 different species. The barcode sequences are mainly taken from the Kingdom Animalia, the Phylum Chordata and belong to the commonly know paraphyletic group of the fishes. In this case, each sequence is extended to 675 sites (or nucleotides) with special handling for gaps, if necessary. Because of the different haplotypes and the distinct Mitochondrial Regions the lengths of the sequences evidently differ from each other. We normalized the downloaded sequences by excluding the under represented species, in order to obtain a balanced training set. For this data set the class distribution is fair, with 8 specimens for each species, on the average.

Adopting the previously described scheme, we obtain the results presented in Table 6 and in Table 7.

**Table 6:** Optimal values and Error Rates (third data set)

			**Error Rates**	
***β***	***α***	**test%**	**training**	**testing**
15	5	10	4.78%	7.06%
15	6	10	4.78%	8.74%
15	6	20	3.30%	10.64%
15	5	20	10.52%	17.73%

average			5.84%	11.04%

20	7	10	4.02%	4.75%
20	8	10	1.15%	9.71%
20	8	20	8.25%	14.18%
20	7	20	10.93%	16.31%

average			6.08%	11.23%

25	10	10	0%	3.78%
25	10	10	1.53%	8.74%
25	10	20	1.24%	7.80%
25	10	20	1.65%	5.67%

average			1.10%	6.49%

**Table 7:** Logic Formulas for Species 1 to 5 (third data set)

**SPECIES**	**CC**	**WC**	**CLAUSE(S)**
Ompok bimaculatus	1.00	0.00	(v400 = t) and (v556 = t) and (v607 = c)
Ompok pabo	1.00	0.00	(v287 = a) and (v329 = a)
Glyptothorax ventrolineatus	1.00	0.00	(v36 = g) and (v267 = a) and (v308 = g) and (v589 = c)
Glyptothorax brevipinnis	1.00	0.00	((v219 = a) and (v408 = t)
Parambassis ranga	1.00	0.00	(v545 = g) and (v556 = a) and (v607 = a)

The results shown in Table 6 are related to the last analysis, in which we have used a higher number of features (15, 20 and 25) in order to obtain similar results to the previous data sets (as shown in Table 2 and 4). The formulas are still very compact (see Table 7) and the recognition rates are in line with those of the other experiments.

Some general considerations on the results obtained are:

• the number of sites used for identifying the class of an individual is always very small - smaller, in general, of what is obtained in other experiments presented in the literature (e.g., in [18]), confirming the validity of the barcode information to discriminate among species;

• the precision of the formulas, when evaluated on a training-test split of the data, is always very high and in line with previous results; even if the method needs a sufficient number of specimens as representative of a species in order to provide robust formulas, a small number (near 10) seems to be sufficient to obtain good results (as shown in particular by the third data set examined);

• the formulas produced by the proposed method are not constrained to form a taxonomic structure of the analyzed species (differing from other clustering-oriented methods as Neighbor Join [20]).

• the problem sizes of the described experiments have not caused any computational problem to our method. The feature selection model is linear in the number of specimens, while the dimensions of the barcode fragment (below 700 sites) is not at all a problem for standard integer programming solvers. When the number of specimens rises in size, then the use of a specifically designed GRASP algorithm (see [17]) keeps the solution time in a linear relation with the problem size. On the other hand, the identification of the logic formulas with *Lsquare*, although very fast with the considered dimensions, may require longer times when the number of specimens ranges in the tens of thousands. For this reason we are presently experimenting more efficient and parallel implementations of a solution algorithm that would possess, for this part of the method, the desirable scalability.

## Conclusion

In this work we have discussed the application of Data Mining methods for specimen classification. We consider the problem of the analysis of barcode - a particular fragment of mitochondrial DNA that has recently been identified as a potential collector of genetic information that is useful to discriminate among species. The method adopted is comprised of two main steps; the first is based on the compression of the barcode into a reduced set of very informative features using a particular integer programming formulation; the second consists in the application of a logic mining method - the *Lsquare *system - to identify separating formulas on the compressed data. The method appears to be practical, sufficiently fast and precise, exhibits small error rates and produces extremely compact separating formulas for the data sets considered in the experiments. Such latter feature plays a very important role in this type of applications as it results in consistent semantic value that can be used by field experts to enhance and complete their knowledge of the studied phenomenon - in this case, the relation between species taxonomies and the COI mitochondrial DNA.

The results described in this paper direct the interest of future research into two main directions that go beyond the scope of this paper: one is the detailed comparisons of the correct recognition results with other similar character-based methods with specifically designed experimental designs; the other one is the study of the relations among the small classification formulas obtained and the taxonomical tree-like strucures that are present in the data.

## Competing interests

The authors declare that they have no competing interests.

## Authors' contributions

The method described was originally adapted to barcode analysis by Paola Bertolazzi and Giovanni Felici. Software design and implementation was performed by Giovanni Felici and Emanuel Weitschek. Experiments were designed and conducted by Giovanni Felici and Emanuel Weitschek. All authors contributed equally to the analysis of the experiments and to the writing of the paper.
